# Single session removal of encrusted double J stent using a simultaneous endourological approach: A case series

**DOI:** 10.1097/MD.0000000000046323

**Published:** 2026-05-12

**Authors:** Zoltán Kiss, Gyula Drabik, Mihály Murányi, Attila Nagy, Ioannis Kartalas Goumas, Tibor Flaskó

**Affiliations:** a Department of Urology, University of Debrecen, Debrecen, Hungary; b Department of Health Informatics, Faculty of Health Sciences, University of Debrecen, Debrecen, Hungary; c Clinical Institute Beato Matteo, Vigevano, Italy.

**Keywords:** cystolitholapaxy, encrusted double J stent, PCNL, ureteroscopy

## Abstract

Double J (DJ) stent encrustation is a serious urological complication that often requires complex surgical management. This study evaluated the feasibility, safety, and efficacy of a novel combined antegrade and retrograde endourological approach to remove encrusted DJ stents. A retrospective analysis was conducted involving 12 patients treated between May 2020 and April 2025 at a tertiary university hospital. All patients underwent simultaneous antegrade and retrograde procedures. The inclusion criterion was encrustation of both the renal and vesical coils, with some cases involving ureteral encrustation. The visual grading for the ureteral encrusted stent classification system was used to evaluate surgical complexity. Postoperative outcomes, complications, and stone-free rates were assessed. The mean age of patients was 41.17 ± 20.74 years, with a mean stent indwelling time of 17.08 ± 12.80 months. The average operative time was 72.92 ± 45.90 minutes. All procedures were performed using a single-tract access, and all patients became stent-free within 1 session. Tubeless percutaneous nephrolithotomy was performed in 11 patients; 1 patient had a Clavien–Dindo grade II complication. Otherwise, no major complications (grades III–V) were observed. The mean decrease in hemoglobin level was statistically significant yet clinically negligible, and no transfusions were required. The simultaneous combined endourological approach in the supine position is feasible, effective, and safe for removing encrusted DJ stents. Performing antegrade and retrograde procedures concurrently by 2 surgeons is efficient as it reduces the operative time and potentially minimizes healthcare burden. Although promising, further prospective studies are warranted to confirm these findings and establish broader clinical applicability.

## 1. Introduction

Double J (DJ) stents are routinely used worldwide due to their versatile applications in daily urological practice, including relieving ureteral obstructions, facilitating urine drainage following endourological procedures, and serving as pre-stents to dilate the ureter.^[1]^ Nonetheless, these stents can cause complications such as hematuria, dysuria, migration, fragmentation, encrustation, and recurrent urinary tract infections.^[2]^ The broader application of DJ stents has consequently increased the prevalence of associated adverse events. Among these complications, encrustation is a serious issue affecting both adults and children.^[3]^ Managing encrusted DJ stents is challenging and time consuming; it can increase the burden on healthcare systems as treatment costs are estimated to be almost 7 times higher compared to timely DJ stent removal.^[4]^ To extract DJ stents, several techniques may be employed, including extracorporeal shockwave lithotripsy, transurethral or percutaneous cystolitholapaxy (TUCL), ureteroscopy (URS), flexible ureterorenoscopy, and percutaneous nephrolithotomy (PCNL). Open surgery is also a viable option.^[5]^

Traditionally, the aforementioned endourological procedures were performed over multiple sessions or in a single session by a single surgeon in a metachronous way,^[6,7]^ and our literature review revealed only a few reports on the use of a combined endourological approach for DJ stent removal performed simultaneously by 2 surgeons.^[8,9]^ Therefore, our objective was to highlight the feasibility and benefits of a combined endourological approach for DJ stent removal, a method which we hypothesized would offer potential advantages in terms of efficiency.

## 2. Methods

Between May 2020 and April 2025, 12 patients underwent encrusted DJ stent removal through a simultaneous endourological approach at a tertiary university hospital. This research was approved by the Regional Institutional Review Board (IRB no. DERKEB/IKEB 7161-2025). Written informed consent was obtained from all patients before data collection. Information identifying patients was removed, after which the authors no longer had access to personal data, and the subsequent analysis was performed anonymously. Of the 12 patients, 5 were referred from other institutions. The inclusion criterion for the study was encrustation of both the renal and vesical coils of the DJ stent, with 2 patients also exhibiting encrustation of the ureteral portion. Exclusion criteria were nonfunctioning kidneys (<20% function on renal scintigraphy) and unresolved urinary tract infections. All interventions were conducted by the same surgical team, ensuring consistency in patient management. All antegrade interventions were carried out by a single surgeon with expertise in PCNL, whereas the retrograde interventions were performed by another surgeon specialized in flexible ureterorenoscopy.

**Figure 1.**
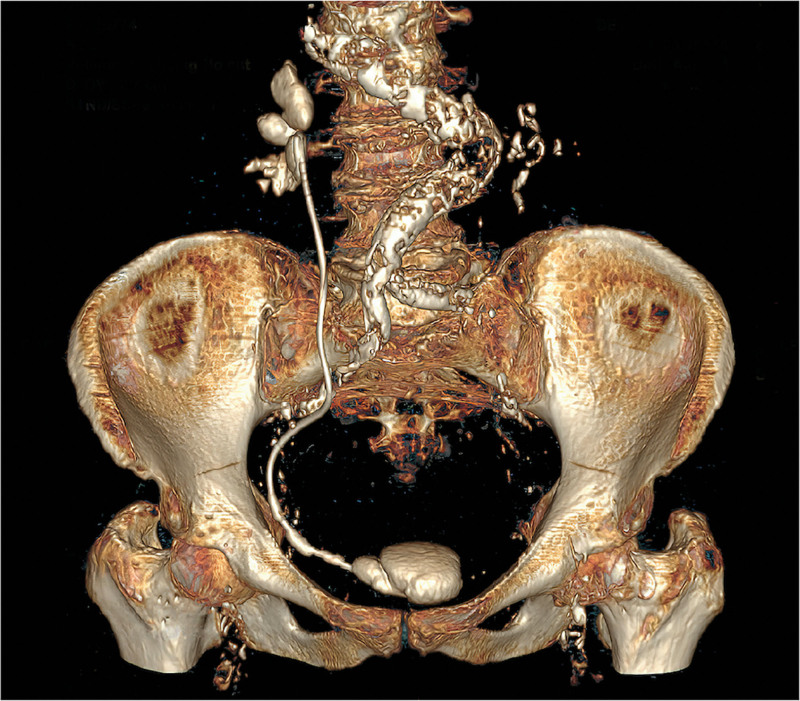
Three-dimensional reconstruction of CT images showing an encrusted DJ stent. CT = computed tomography, DJ = double J.

Patient characteristics such as age, sex, body mass index, and American Society of Anesthesiologists scores were evaluated. Each patient underwent urinary tract ultrasound examination, kidney–ureter–bladder (KUB) radiography, and low-dose abdominal and pelvic computed tomography (Fig. 1). Indications for DJ stent insertion, causes of encrustation, and indwelling time were documented. The indwelling time was determined from the time of insertion of the DJ stent to the date of removal. To predict the complexity of surgery, a visual grading system for ureteral encrusted stent was applied.^[10]^ In addition, urine culture results and relevant laboratory values, including serum creatinine and hemoglobin concentrations, were assessed.

### 2.1. Description of the surgical method

**Figure 2.**
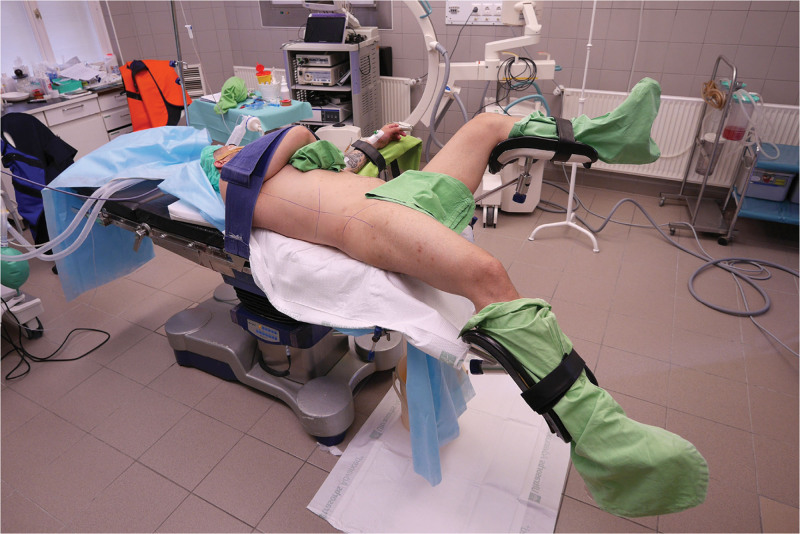
Barts ”flank-free” modified supine position.

**Figure 3.**
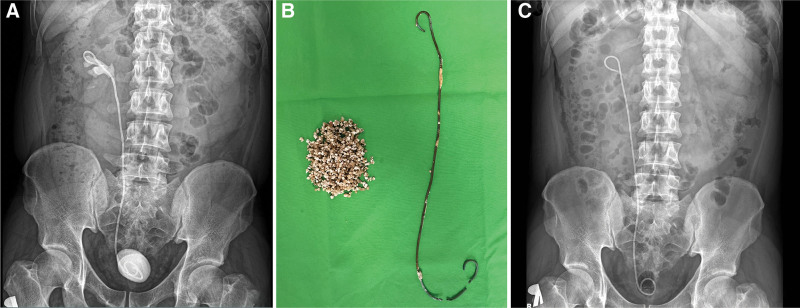
Removal of encrusted DJ stent using a combined endourological approach. (A) KUB radiograph of an encrusted DJ stent. (B) Removed encrusted DJ stent. (C) Postoperative KUB radiograph. DJ, double J; KUB, kidney–ureter–bladder.

Intravenous antibiotics were administered to the patients 30 minutes before the start of surgery. Typically, a broad-spectrum third-generation cephalosporin, such as ceftriaxone (2 g), was used. In cases of positive preoperative urine culture, targeted parenteral antibiotic therapy (most commonly a cephalosporin) was initiated the day before surgery and continued during hospitalization. Under general anesthesia, patients were positioned in supine position with a 15° to 20° lateral tilt, ensuring careful padding and protection of pressure points (Fig. 2). The ipsilateral leg remained extended, while the contralateral leg was flexed. Key anatomical landmarks, such as the posterior axillary line, rib line, and iliac crest, were marked on the patient. Cystoscopy was performed to identify the ipsilateral ureteral orifice and to visualize the encrusted DJ stent. Subsequently, a ureteral catheter was placed into the ipsilateral ureter alongside the encrusted DJ stent. Beside the ureteral catheter, a 25-Fr nephroscope (Olympus Winter & IBE GmbH, Hamburg, Germany) was inserted, and a ShockPulse lithotripter was used by the retrograde surgeon for TUCL. During lithotripsy of the encrustation of the vesical coil of the DJ stent, the antegrade surgeon evaluated the perirenal anatomy using ultrasonography (Mindray Diagnostic Ultrasound System, Consona N9, Shenzhen, China). After selecting the target calyx, an 18-gauge needle (Cook Incorporated, Bloomington, IN) was inserted under the posterior axillary line using X-ray guidance. A 0.035-mm hydrophilic guidewire (Roadrunner, Cook Incorporated, Bloomington, IN) was then advanced into the ureter. Renal tract dilatation was accomplished incrementally under fluoroscopy using a teflon renal dilator set (Cook Inc., Bloomington, IN) in 3 steps: 14-, 20-, and 28-Fr. Thereafter, a 28-Fr Amplatz sheath and a 25-Fr nephroscope (Olympus Winter & IBE GmbH, Hamburg, Germany) were employed. The guidewire via the renal access sheath ensured safety throughout the procedure. Lithotripsy of the encrustation of the renal coil of the DJ stent and kidney stones was performed using an ultrasound lithotripter (ShockPulse-SE Lithotripsy System; Cybersonics Inc., PA). Once simultaneous lithotripsy was completed, the DJ stent was gently removed transurethrally using fluoroscopy (Fig. 3).

In cases wherein the ureteral part of the DJ stent was encrusted, a 0.035-mm hydrophilic-coated guidewire (Roadrunner, Cook Incorporated, Bloomington, IN) was advanced into the ureter by the retrograde surgeon after TUCL. An access sheath was not placed in the ureter due to an encrusted DJ stent. A 7.5-Fr disposable flexible ureteroscope (Scivita Medical Technology Co., Ltd., Suzhou, China) was introduced into the ureter along with the DJ stent. For lithotripsy of the ureteral encrustation on the DJ stent, a 30-W Ho:YAG laser (Quanta System S.p.A., Samarate, Italy) with a 272-μm laser fiber was used at a maximum power of 10 W. At the end of the procedure, a 26- or 28-cm long 6-Fr DJ stent (Urotech GmbH, Achenmühle, Germany) was placed by the retrograde surgeon. The urinary catheter and DJ stent were withdrawn after 1 day and 1 week, respectively.

### 2.2. Assessment of surgical results

The duration of surgery was manually recorded. The number and anatomical location of percutaneous tracts, method of urinary drainage, and duration of hospitalization were documented. On the first postoperative day, hemoglobin and serum creatinine levels were measured, and KUB radiography was performed to verify the DJ stent placement. Complications arising within 30 days after surgery were classified according to the Clavien–Dindo grading system. Stone-free rates were evaluated at 1 month postoperatively using KUB radiography and ultrasonography.

### 2.3. Statistical analysis

Normality of data distribution was assessed using the Shapiro–Wilk test. Continuous variables were described as means and standard deviations. Paired *t* tests were used to compare preoperative and postoperative values and to assess statistical significance. Statistical analyses were conducted using Intercooled Stata v18.0. (StataCorp. 2023, Stata Statistical Software: Release 18. StataCorp LLC, College Station, TX), with statistical significance set at *P* < .05.

## 3. Results

Table 1 summarizes the demographic and clinical characteristics of the study participants. The mean age of the participants was 41.17 ± 20.74 years, and the mean body mass index was 25.91 ± 10.39 kg/m^2^. The mean indwelling time of DJ stents was 17.08 ± 12.80 months. Preoperative urine cultures were positive in 50% of the participants. *Escherichia coli* was identified in 4 cases, *Proteus mirabilis* in 1 case, and *Staphylococcus aureus* in 2 cases. The mean stone density was 998.83 ± 382.85 HU. The average operative time was 72.92 ± 45.90 minutes. All procedures were conducted using a single percutaneous tract during supine PCNL. In 10 patients, access was obtained through the lower calyx, whereas in 2 patients, access was obtained via the middle calyx. In 2 cases, the distal coil was covered by a large stone burden, making the ureteral orifice inaccessible. In this situation, we first fragmented the stone transurethrally next to the ureteral orifice to create access. Thereafter, the ureteral catheter could be inserted, allowing the percutaneous surgeon to start the procedure simultaneously. For urinary diversion, the tubeless technique (using only the DJ stent) was used in 11 patients. One patient with a solitary kidney required both a DJ stent and a nephrostomy tube for safety reasons. The mean hemoglobin decrease was 12.25 ± 2.88 g/L, which was statistically significant (*P* = .001) but clinically insignificant. None of the patients required blood transfusion. The mean decrease in serum creatinine levels was − 10.83 ± 17.63 µmol/L, which was not statistically significant (*P* = .057). The mean pre- and postoperative GFRs were 73.67 ± 22.17 and 64.83 ± 21.97, respectively (*P* = .014). All patients were stent-free after a single session. Regarding stone-free rates, 11 patients were stone-free. In 1 patient, a 6-mm residual fragment in the lower calyx was detected on KUB radiography during the 1-month follow-up period. All patients were discharged 1 day following surgery. In terms of complications, 1 patient was readmitted due to pyelonephritis and fever, which required treatment with parenteral antibiotics (Clavien–Dindo grade II). No Clavien–Dindo grades III, IV, or V complications were reported.

**Table 1:** Demographic and clinical characteristics of the study population.

Patient	Sex	Laterality	ASA score	Symptoms	History of urolithiasis	V-GUES score	Reason of DJ stent encrustration	DJ stent indwelling time (month)	Type of surgeries
1	Male	Right	2	Flank pain	Yes	C	Negligence	8	Supine PCNL, TUCL
2	Female	Left	1	Flank pain	No	C	Pregnancy	15	Supine PCNL, TUCL
3	Male	Left	3	Flank pain	Yes	D	Negligence	6	ECIRS, TUCL
4	Female	Right	3	Flank pain, acute cystitis	No	C	Postponed URS due to the COVID 19 pandemic	15	Supine PCNL, TUCL
5	Female	Right	1	Hematuria	No	C	Postponed URS due to the COVID 19 pandemic	8	Supine PCNL, TUCL
6	Female	Left	3	Fever, flank pain	Yes	D	Negligence	31	ECIRS, TUCL
7	Female	Right	2	Flank pain	Yes	D	Postponed URS due to the COVID 19 pandemic	6	ECIRS, TUCL
8	Female	Left	3	Flank pain	Yes	C	Postponed URS due to the COVID 19 pandemic	32	Supine PCNL, TUCL
9	Female	Right	1	Asymptomatic	No	C	Pregnancy	14	Supine PCNL, TUCL
10	Male	Right	2	Asymptomatic	Yes	D	Negligence	11	ECIRS, TUCL
11	Female	Left	2	Asymptomatic	No	C	Physician’s poor communication after ureteral neoimplantation	12	Supine PCNL, TUCL
12	Male	Right	1	Flank pain, dysuria, hematuria	Yes	C	Negligence	47	Supine PCNL, TUCL

ASA = American Society of Anesthesiologists, DJ = double J, ECIRS = endoscopic combined intrarenal surgery, PCNL = percutaneous nephrolithotomy, TUCL = transurethral cystolitholapaxy, URS = ureteroscopy, V-GUES = visual grading for ureteral encrusted stent.

## 4. Discussion

Ureteral stenting has been part of the surgical armamentarium for nearly 6 decades, revolutionizing urological practice by providing an effective solution for managing ureteral obstruction.^[11]^ DJ stents, developed by Finney in 1978, have become widely accepted and increasingly used for urinary diversion.^[12]^ DJ stents are integral components of various urological procedures, highlighting their importance in modern urological care. DJ stents are also used in cases of extrinsic or intrinsic ureteral obstruction, during abdominal or gynecological surgeries to aid ureter identification, and as a scaffold after reconstructive urological procedures such as pyeloplasty, ureteral reimplantation, or renal transplantation.

Despite their advantages, DJ stents carry a risk of complications, with encrustation being the most severe. A study by Ali et al, involving 778 patients, reported an encrustation prevalence rate of 20.7%.^[13]^ Risk factors for encrustation include a long indwelling time, poor patient compliance, pregnancy, and a history of urolithiasis and pyelonephritis.^[14]^ Of these, indwelling time is the most important. Three months after DJ stent placement, the probability of encrustation significantly increases to 76.3%.^[15]^ Interestingly, frequent stone formers have a higher risk of renal coil encrustation, whereas urinary tract infections and older age are common risk factors for vesical coil encrustation. This phenomenon can be explained by the storage function of the bladder. Subvesical obstruction can occur in elderly patients, and lower urinary tract infections are more common than upper ones.^[16]^ The mechanism of encrustation is complex and involves bacterial biofilm formation on the stent surface and elevated mineral levels in the urine.^[1]^ Currently, there are no clear guidelines for managing encrusted DJ stents, although some authors have proposed treatment algorithms.^[1,6–8]^ Removing encrusted DJ stents presents a unique challenge for urologists and requires meticulous planning, a wide range of instruments, and thorough patient counseling. Removing encrusted DJ stents forcibly is not recommended because it can lead to ureteral avulsion or stent fragmentation. If the DJ stent cannot be easily removed following lithotripsy, reevaluation is necessary. The DJ stent should be carefully inspected along its entire length to ensure no residual encrustation remains. Because of the risk of fragmentation, it is essential to verify that all segments of the DJ stent are removed. Even if only the vesical coil of the DJ stent is encrusted, its removal should not be forced as the adhered stone can injure the urethra, increasing the risk of urethral stricture.

Treatment depends on the severity and location of the encrustation and preference of the surgeon. Various scoring systems, such as the KUB and forgotten, encrusted, calcified (FECal) grading systems, have been used to predict the complexity of surgery.^[17]^ The KUB grading system quantifies the stone burden separately in the kidney, ureter, and bladder segments of an encrusted stent, assigning scores from 1 to 5 according to the maximum diameter of the encrustation.^[18]^ Similarly, the FECal grading system classifies stent encrustation severity from grade 1 to 5, based on stone size, location, and extent of encrustation.^[19]^ In this retrospective study, we used the visual grading for ureteral encrusted stent classification system described by Manzo et al, which predicts the number of necessary procedures.^[10]^ The authors described 4 groups (A–D) of encrustation based on preoperative non-contrast computed tomography and concluded that an endoscopic combined approach provided the best stent- and stone-free rates in cases of severe encrustation (types C and D stents).

If prone PCNL is chosen, lithotripsy of the encrustation on the vesical coil of the DJ stent must first be performed. In cases where ureteral encrustation is also present, an additional URS or flexible URS procedure is required after TUCL. Subsequently, the patient must be repositioned and resterilized for prone PCNL. This approach significantly increases operative time and procedural complexity. Occasionally, TUCL and URS procedures may become prolonged, potentially compromising patient safety if prone PCNL is performed in the same session. Therefore, the prone PCNL is usually scheduled for a second session.

The introduction of supine PCNL by Valdivia was a major milestone in stone surgery as it allowed both antegrade and retrograde approaches in the same session.^[20]^ The optimal patient positioning, particularly comparing prone and supine approaches, remains debatable among urologists performing PCNL. While each positioning technique offers distinct advantages, we believe the supine position is preferable in scenarios involving severely encrusted DJ stents with extensive encrustation in both the renal and vesical coils. A combined endourological approach can facilitate the removal of encrusted DJ stents, highlighting the advantages of utilizing the supine position. We believe that the duration of surgery can be shortened if 2 surgeons perform DJ stent removal simultaneously rather than sequentially, especially in cases of large stone burden. Reducing the operative time is crucial because it can potentially lower the complication rate. Moreover, simultaneous procedures likely decrease anesthesia duration, associated healthcare costs, and patient discomfort, highlighting further practical benefits.

Animal studies have demonstrated that DJ stents, particularly with prolonged indwelling time, may adversely affect ureteral peristalsis, necessitating a longer duration for functional recovery. Consequently, we emphasize the importance of scheduling a follow-up visit after removing an encrusted DJ stent, during which ultrasonography should be performed to detect any potential silent hydronephrosis, alongside assessing renal function parameters.^[21,22]^

We hypothesized that a combined endourological approach for DJ stent removal can offer potential advantages in terms of efficiency. In our case series, all patients achieved a stent-free status, demonstrating the feasibility and effectiveness of the single-session combined approach. Given the technical complexity of these procedures, preoperative counseling is of utmost importance. Patients are informed that the interventions are technically challenging and that removal of the DJ stent may not be possible in a single session. We also emphasize the importance of close postoperative follow-up. Nevertheless, we agree that prevention is the optimal treatment option. Proper indications for DJ stent insertion are the cornerstone of prevention. Comprehensive patient counseling, close follow-up, and the use of computerized tracking programs can decrease the encrustation rate.^[23]^ This issue is also important from a legal perspective as up to 16% of endourology-related lawsuits are associated with retained stents.^[24]^ In our series, no legal actions were encountered. The limitations of our study include its single-center retrospective design and small patient cohort. Further studies are required to confirm the broad applicability of this method.

Our case series underscores the feasibility and efficacy of combined antegrade and retrograde endourological approaches for removing encrusted DJ stents. The use of this method in the supine position demonstrates its versatility, allowing both techniques to be performed simultaneously and thereby reducing the overall operative time. The effectiveness of this combined method, along with its potential to reduce the healthcare burden by performing both procedures in a single session, highlights its value in clinical practice. Nevertheless, the prevention of encrustation remains paramount, and proper patient selection, comprehensive counseling, and regular follow-ups are crucial to minimize the risk of stent encrustation.

## Acknowledgments

We would like to acknowledge the support of Editage (www.editage.com) in manuscript preparation.

## Author contributions

**Conceptualization**: Zoltán Kiss.

**Formal analysis**: Attila Nagy.

**Methodology**: Zoltán Kiss.

**Supervision**: Tibor Flaskó.

**Writing – original draft**: Zoltán Kiss.

**Writing – review & editing**: Gyula Drabik, Mihály Murányi, Ioannis Kartalas Goumas, Tibor Flaskó.
